# Comprehensive elucidation on the genetic profile of the Hezhou Han population *via* an efficient InDel panel

**DOI:** 10.1093/fsr/owae021

**Published:** 2024-04-09

**Authors:** Shuyan Mei, Wei Cui, Man Chen, Meiming Cai, Fanzhang Lei, Xi Wang, Shangwu Yang, Bofeng Zhu

**Affiliations:** Key Laboratory of Evidence Science (China University of Political Science and Law), Ministry of Education, Beijing, China; College of Basic Medicine and Forensic Medicine, Henan University of Science and Technology, Luoyang, China; Guangzhou Key Laboratory of Forensic Multi-Omics for Precision Identification, Department of Forensic Genetics, School of Forensic Medicine, Southern Medical University, Guangzhou, China; Guangzhou Key Laboratory of Forensic Multi-Omics for Precision Identification, Department of Forensic Genetics, School of Forensic Medicine, Southern Medical University, Guangzhou, China; Guangzhou Key Laboratory of Forensic Multi-Omics for Precision Identification, Department of Forensic Genetics, School of Forensic Medicine, Southern Medical University, Guangzhou, China; Guangzhou Key Laboratory of Forensic Multi-Omics for Precision Identification, Department of Forensic Genetics, School of Forensic Medicine, Southern Medical University, Guangzhou, China; Guangzhou Key Laboratory of Forensic Multi-Omics for Precision Identification, Department of Forensic Genetics, School of Forensic Medicine, Southern Medical University, Guangzhou, China; The People’s Hospital of Hezhou, Hezhou, China; Key Laboratory of Evidence Science (China University of Political Science and Law), Ministry of Education, Beijing, China; Guangzhou Key Laboratory of Forensic Multi-Omics for Precision Identification, Department of Forensic Genetics, School of Forensic Medicine, Southern Medical University, Guangzhou, China; Key Laboratory of Shaanxi Province for Craniofacial Precision Medicine Research, College of Stomatology, Xi’an Jiaotong University, Xi’an, China

**Keywords:** InDel, Hezhou Han population, genetic relationship, cross-validation, ancestry information inference

## Abstract

The Han nationality is widely distributed in different regions, and it is one of the most populous nationalities in China. Compared with the ethnic minorities in Guangxi Zhuang Autonomous Region, there is relatively less research on Han individuals dwelled in Guangxi as a part of Chinese Han population. In this study, the genetic polymorphisms of 57 autosomal insertion/deletion (InDel) loci were explored in Hezhou Han (HZH) population. Forensic-related parameters revealed that these 57 InDel loci had high forensic validity and could be used in forensic practice application. In addition, the genetic relationships between the HZH population and 30 worldwide reference populations were explored using a variety of analytical methods, such as phylogenetic tree, principal component analysis, and genetic structure analysis. These results demonstrated that there were closer genetic relationships between the HZH and nine populations from East Asia (EAS). The prediction accuracy rates of five inter-continental cross-validation analyses for individuals from EAS was >0.9, and the prediction accuracy rates of three inter-continental cross-validation analyses for individuals from EAS, Europe, and Africa were all >0.95. In addition, 24 of the 57 InDel loci could be served as ancestral information inference loci, which could effectively distinguish individuals of EAS, Europe, and Africa. In conclusion, these InDel loci could be used not only as a good tool for individual identification and paternity testing in HZH population, but also as an auxiliary tool for ancestry information inference research.

## Introduction

Currently, the two most important tasks in forensic genetics are individual identification and paternity testing. Short tandem repeat (STR) genotyping technology based on the polymerase chain reaction–capillary electrophoresis (PCR–CE) platform is the most important detection method for individual identification and paternity testing in forensic DNA laboratories around the world. However, as the means of crime become more and more intelligent, it is also increasingly difficult to examine biological materials found at crime scenes, and the role of STR loci has exposed more and more deficiencies in the actual inspection process. For example, the commonly used STR loci with long amplicon fragments have limited detection ability for degraded samples; the number of STR loci in the human genome limits the number of candidate loci which do not meet the high requirement for forensic application; in addition, the relatively high mutation rate is not conducive to the interpretation of complex kinship result [1]. Insertion/Deletion (InDel) is a length polymorphism genetic marker formed by the insertion or deletion of DNA fragments of different lengths. It is widely distributed in the genome [2], and it is known that there are approximately 8.8 million InDel loci in the human genome [3]. The InDel loci have the advantages of short amplicon fragments and low mutation rates [4]. In addition, the InDel locus, as a genetic marker of length polymorphism, can be detected using PCR–CE platform [5, 6]. As an ideal genetic marker, the InDel locus plays an indispensable role in challenging cases in forensic practice.

Since the first human genome InDel variation map was successfully drawn in 2006 [7], researchers discovered that it plays an important role in the fields of molecular biology and genetics. Many scholars have used the multiple amplification systems constructed using InDel loci to study the genetic structures of different populations. Pereira et al. [8] screened and analyzed 38 polymorphic InDel loci with amplicon fragments of <160 bp in 306 individuals from Eurasia and Africa (AFR). The results indicated that the 38 InDels were polymorphic and could be used for forensic individual identification. Li et al. [9, 10] successfully constructed a panel including 29 InDel loci for the purpose of forensic application, and the results proved that these 29 InDels were useful in the individual identifications of Chinese populations, but they were poor in paternity testing. Invastigator DIPplex (Qiagen, Hilden, Germany) is a commercial kit capable of amplifying 30 InDel loci simultaneously [11]. Several studies have demonstrated that the kit has high efficacy for forensic applications in some populations [12–17]. However, previous studies have shown that the individual identification power of the kit is weaker in Chinese populations than in Europe (EUR) populations [18]. In addition, AGCU ScienTech Incorporation (Wuxi, China) constructed the AGCU InDel 50 panel for forensic application in Chinese populations, which included 47 autosomal InDel loci, two Y-InDel loci, and one Amelogenin locus. Compared with the Investigator DIPplex panel, this kit has improved genetic polymorphisms of loci, and can obtain more complete and reliable genotype profiling in degraded sample [19]. The research results of the AGCU InDel 50 panel in Chinese several populations revealed that although the panel can be used as a tool for individual identification, it cannot meet the need of paternity testing [19–23]. Therefore, based on the panel, eight low polymorphic loci were removed, and 18 high polymorphic InDel loci were added to form a new AGCU InDel 60 panel (60 InDel panel). This new kit has been proven to be highly sensitive, robust, accurate, and species-specific, and can be used as an effective tool in forensic practices [24]. At present, there are few studies using the 60 InDel panel in Chinese populations. The research of genetic polymorphisms in more populations in China not only helps to understand the genetic backgrounds of different ethnic groups, but also helps to expand the genomic DNA database.

The Han nationality is the largest of the 56 ethnic groups in China, and it is widely distributed in different provinces. It is of great significance to study the InDel genetic polymorphisms of the Han populations from different geographical regions and to explore their genetic relationships with other reference groups. The Guangxi Zhuang Autonomous Region is one of the five autonomous regions in China. It is one of the settlements of ethnic minorities in China, and also one of the provinces with the largest population of ethnic minorities. Most researchers have previously conducted studies on various genetic markers of ethnic minorities living in Guangxi province [25–30], but few studies on the Han population in Guangxi province have been reported. This study is intended to use the 60 InDel panel to reveal the genetic characteristics of 206 healthy unrelated volunteers of the Han nationality in Hezhou city, Guangxi province, and explore their genetic relationships among Hezhou Han (HZH) and 30 worldwide reference populations, mainly including the previously published Dingjie Sherpa (SP) [31], Yunnan Miao (YNM) [32], Hainan Li (HNL) [33], Hunan Han (HNH) [34], and 26 populations from five different geographic continents downloaded from the Ensemble database (https://asia. ensembl.org/index.html).

## Methods and materials

### Sample collection and DNA extraction

The blood samples were collected from 206 healthy volunteers in the Han population in Hezhou city, Guangxi province, after obtaining their written informed consents, and then smeared on the FTA card to prepare a bloodstain sample. All volunteers were unrelated within three generations. Each 3 mm^2^ bloodstain sample was prepared and placed in a 1.5 mL Eppendorf tube. The Chelex 100 method was used to extract DNA from bloodstain samples. This research was approved by the Ethics Committee of Xi'an Jiaotong University (Ethical approval number: 2019–1039), and all procedures followed the experimental operating rules and standards of the Southern Medical University and Xi'an Jiaotong University. Deionized water and DNA 9948 were used as negative and positive controls in this experiment, respectively.

### PCR amplification and InDel genotyping

The 60 InDel panel is a multiplex amplification system that uses six-colour fluorescent labels, and it can simultaneously amplify and detect 57 autosomal InDels (57 InDels), 2 Y-InDels, and a sex-determining gene (Amelogenin) in one reaction [24]. PCR reactions and sequence were consistent with previous study [24]. Allelic genotyping was performed using GeneMapper® ID-X software v1.6 (Thermo Fisher Scientific, Foster City, CA, USA) with a reference peak height threshold of 100 relative fluorescence units (RFUs).

### Statistical analysis

The allelic frequencies, forensic-related parameters, and pairwise linkage disequilibrium (LD) analyses of 57 InDel loci were calculated by STRAF online software [35] (http://cmpg.unibe.ch/shiny/STRAF/). Arlequin software [36] was used to calculate observed heterozygosity (Ho) and expected heterozygosity (He). Hardy–Weinberg equilibrium (HWE) analyses and pairwise *F*_ST_ values among HZH and 30 reference populations (Supplementary Table S1) based on the genotyping data were performed by Arlequin software as well. The Dispan software (https://dispan.com.br/dpcad/) was used to count pairwise *D*_A_ values on the basis of allelic frequencies of 57 InDel loci in HZH and 30 worldwide populations. A neighbour-joining tree (N-J tree) and a circular phylogenetic tree were constructed using MEGA software [37] and itol online software (https://itol.embl.de/itol.cgi) based on the above *D*_A_ values, respectively. The “FactoMineR” package in the *R* software was used to calculate the Cos2 value for each locus of 57 InDel loci. The “ggplot” package in *R* software was used to perform individual principal component analysis (PCA) and population PCA plots based on the genotyping data and allelic frequency data of the HZH and 30 reference populations, respectively. STRUCTURE software (Pritchard Lab, Stanford University, Stanford, CA, USA) was used to analyse the genetic structures of 31 populations with predefined *K* values from 2 to 6, and 20 runs per *K.* Then the results were uploaded to the online structureHarvest software (https://taylor0.biology.ucla.edu/structureHarvester/) for the prediction of the optimum *K* value, and the individual and population Clumpp files of *K* = 2–6 were downloaded at the same time. The best of 20 runs was then calculated on Clumpp software (http://rosenberglab.bioinformatics.med.umich.edu/clumpp.html) and visualized using Distruct software (https://github.com/KIT-MBS/distruct.git). The online software Snipper (http://mathgene.usc.es/snipper/) was used to calculate the informativeness (*In*) values of 57 InDel loci and perform cross validation between individuals at different intercontinental levels.

## Results

### HWE and LD analyses

No peak was observed for the negative control sample, while the positive control and HZH samples showed intact and clear peaks with peak heights >100 RFUs. All 57 InDel loci were in the HWE states in the HZH population (Supplementary Table S3), indicating that the volunteers selected in this study were representative and could represent the entire HZH population. In LD analyses, all loci are not linked, and pairwise loci located on the same chromosome were in linkage equilibrium state after Bonferroni correction, and then the multiplication principle could be used to calculate the accumulation probability (Supplementary Table S2).

### Allelic frequencies and related forensic parameters

**Figure 1:**
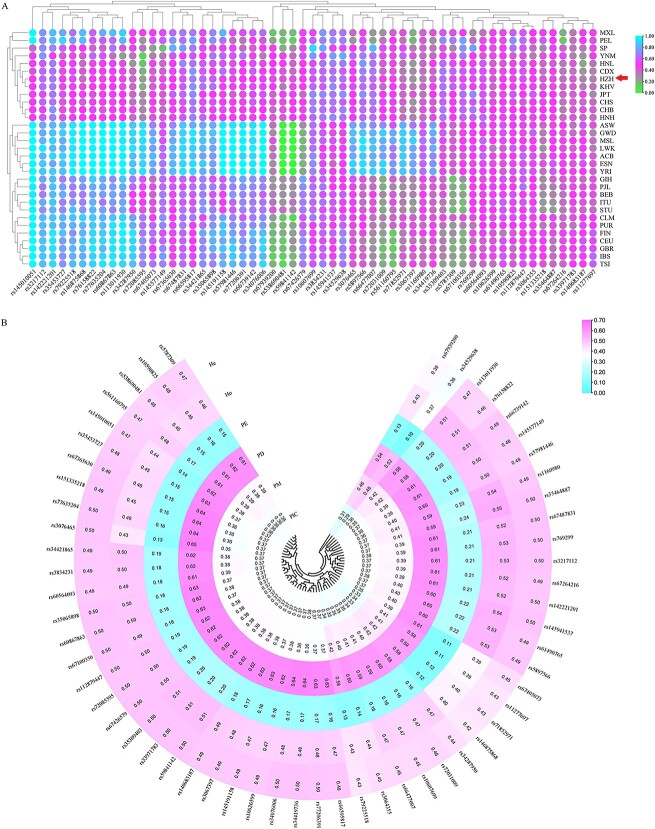
Heatmaps of allelic frequencies and forensic parameters. (A) Insertion allelic frequencies of 57 InDel loci in 31 worldwide populations. (B) Forensic parameters of 57 InDel loci in Hezhou Han population.

Except for three loci whose insertion frequencies were <0.3, the insertion frequencies of the remaining loci were between 0.3 and 0.7 (Supplementary Figure S1). In the heatmap of insertion frequencies of 57 loci in 31 populations (Figure 1A and Supplementary Table S4), colours in the graph from green to purple to blue showed the insertion frequencies from small to large. The allele frequency distributions of 57 InDel loci in the HZH population were relatively uniform (0.3–0.7), which was similar to those of the nine populations from East Asia (EAS), but the allelic frequencies of some loci were different from those in AFR and EUR. These loci with large differences in insertion frequencies could be used as candidate ancestrally inferred loci.

At present, forensic-related parameters mainly include polymorphism information content (PIC), matching probability (PM), power of discrimination (PD), exclusion probability (PE), and heterozygosity (H). The forensic parameter values of 57 InDel loci in the HZH population are shown in Figure 1B and Supplementary Table S3. Among them, the PIC values in the HZH population ranged from 0.291 9 (rs72085595) to 0.375 0 (rs145010051), with an average value of 0.362 4, and PIC values of 10 InDel loci were <0.35. The PM, PD, and PE values were spanned from 0.352 2 (rs5787309) to 0.481 0 (rs72085595), 0.519 0 (rs72085595) to 0.647 8 (rs5787309), and 0.098 8 (rs72085595) to 0.238 6 (rs3076465), respectively, and the mean values were 0.392 5, 0.608 5, and 0.172 5, respectively. He and Ho ranged from 0.355 7 (rs72085595) to 0.501 2 (rs145010051), and 0.373 8 (rs72085595) to 0.553 4 (rs3076465), respectively, with mean values of 0.477 5 and 0.479 5, respectively. The absolute value of the difference between He and Ho at the same locus was >0.05, but <0.1 at four loci. Forensic parameter values in HZH were similar to the nine reference populations from EAS, but the SP and YNM populations had a wider range of PIC, PD, and Ho values in compare with the other nine populations (Supplementary Figure S2 and Supplementary Table S5). The cumulative matching probability (CPM) of the 57 InDel loci in the HZH population was 5.594 5 × 10^−24^, which was smaller than those of other eight populations from EAS, but larger than that of HNH population. The combined exclusion probability (CPE) was 0.999 980 295 in the HZH population, and the CPE values were >0.999 9 in nine populations from EAS. Compared with others from EAS, the CPM and CPE values were larger and smaller in SP and YNM populations, respectively (Supplementary Figure S3 and Supplementary Table S6).

### Genetic distances among 31 populations

The genetic relationships between HZH and the 30 worldwide populations were evaluated by the magnitude of pairwise *F*_ST_ and *D*_A_ values (Supplementary Tables S7 and S8). The range of *F*_ST_ values among HZH and other nine populations from EAS was 0.000 3 to 0.040 8, and there was the smallest *F*_ST_ value between HZH and Kinh in Ho Chi Minh City, Vietnam (KHV) populations, whereas the largest value was between HZH and SP populations. The *F*_ST_ values between HZH and populations from South Asia (SAS), America (AMR), EUR, and AFR ranged from 0.0617 (BEB) to 0.081 9 (GIH); 0.069 7 (MXL) to 0.108 4 (PUR); 0.132 2 (IBS) to 0.138 5 (CEU); 0.172 3 (ASW) to 0.221 9 (YRI), respectively. The pairwise *D*_A_ values among 31 populations ranged from 0.001 0 to 0.099 9. The *D*_A_ values among HZH and the nine popualtions from EAS were the smallest, followed by SAS, AMR, and EUR, while AFR had the largest *D*_A_ values. In EAS populations, the populations with the closest genetic distances to HZH were HNH and KHV, and the farthest genetic distances to HZH was SP*.* In addition, the *D*_A_ values of the HNH population with two populations (Han Chinese in Bejing (CHB) and Southern Han Chinese (CHS)) were both 0.008, which was the smallest *D*_A_ value among pairwise populations in 31 populations.

**Figure 2:**
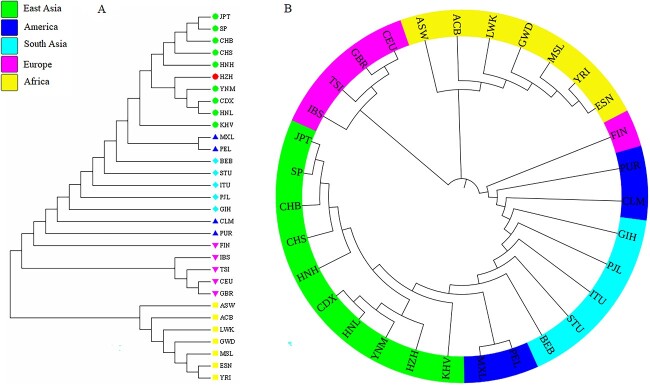
Two phylogenetic trees of 31 different populations. (A) Neighbour-joining tree was performed by MEGA software based on pairwise *D*_A_ values among 31 populations. (B) Circular phylogenetic tree was constructed by iTOL online software on the basis of pairwise *D*_A_ values among 31 populations.

In order to further explore the genetic relationships among 31 populations, two phylogenetic trees were constructed (Figure 2). The N-J tree was divided into two main branches: one was seven populations in AFR, and the other was 24 non-AFR populations. Here, the non-AFR branch was also divided into two sub-branches: one sub-branch was the HZH population and nine populations from EAS, and another sub-branch was the remaining 14 populations. In the latter sub-branch, it can be seen that the genetic distances among different populations from the same continent were relatively close. While the four populations from the AMR were divided into two groups by the SAS populations, one of which was PUR and CLM, and the other was MXL and PEL. A circular phylogenetic tree was performed by iTOL online software (https://itol.embl.de/) on the basis of pairwise *D*_A_ values among 31 populations as well. Populations from the same continent were closer together on the circular phylogenetic tree, which was consistent with the results of the N-J tree.

### PCA and structure analyses

**Figure 3:**
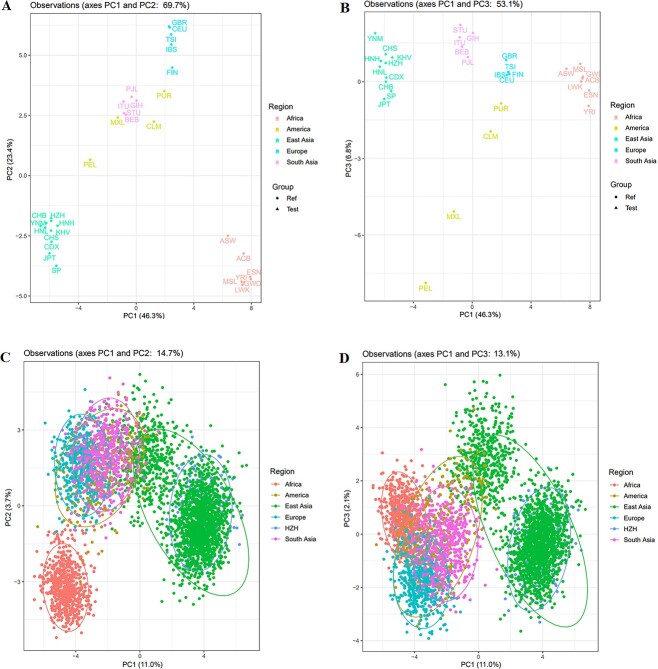
PCA analyses among 31 worldwide populations at population level and individual level. (A) PC1 and PC2 at population level. (B) PC1 and PC3 at population level. (C) PC1 and PC2 at individual level. (D) PC1 and PC3 at individual level.

PCA analysis was used to explore genetic relationships among 31 populations at the population and individual levels, respectively (Figure 3). At population level, the total proportion of PC1, PC2, and PC3 was 76.5%. Populations from EAS and AFR could be distinguished on the PC1 axis; populations from EUR could be distinguished on the PC2 axis; and on the PC3 axis, populations from the AMR could also be distinguished. There were close genetic relationships between the HZH population and nine groups from EAS (Figure 3A and Figure 3B). At the individual level, the total proportion of PC1, PC2, and PC3 was 16.8%. Individuals from the HZH population scattered among populations in EAS. In Figure 3C, individuals from EAS and AFR could be distinguished from the individuals from other continents, and in Figure 3D, only individuals from EAS could be distinguished from those from other continents. It can be seen that the PCA analysis based on 57 InDel loci cannot achieve the effect of distinguishing five continents, which may be caused by the low proportion of the first three principal components at the individual level.

**Figure 4:**
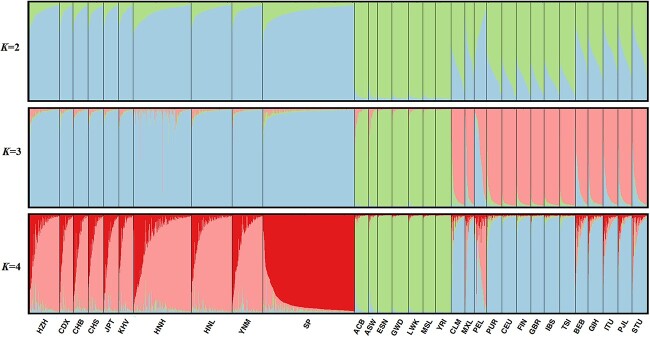
The structure analyses (*K*=2–4) among 31 populations based on the raw genotyping data of 57 InDel loci.

Structure analysis is an analytic method used by most researchers in population genetic research that is used to visualize the genetic compositions of different populations. After evaluation by the online software StructureHarvest, the optimal *K* value was determined to be 3 (Supplementary Figure S4), so we visualized the results of the population structures with *K* values of 2–4 into bar graphs (Figure 4). The HZH population displayed similar ancestral components to the nine populations in EAS. At *K* = 2, three groups (EAS, AFR, and others) of ancestral components could be distinguished. At *K* = 3, four ancestral component groups were displayed, which were EAS; AFR; EUR, CLM, and PUR; and SAS, MXL, and PEL. At *K* = 4, the SP population geographically derived from EAS exhibited different ancestral components with other EAS populations, while the PEL population showed different ancestral components with the AMR populations.

### Cross-validation analyses

**Figure 5:**
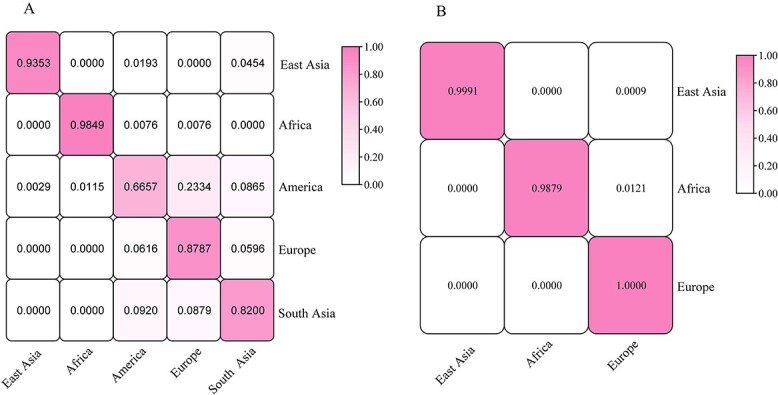
Prediction matrix for different intercontinental populations based on 57 autosomal InDel genotyping data. (A) Cross-validation success rates of 4 224 individuals in five intercontinental populations. (B) Cross-validation success rates of 3 388 individuals in three intercontinental populations.

The success rate of this panel in predicting the origin of the intercontinental population was assessed based on the genotyping data of the 57 InDel loci of the individuals from 31 populations using the “verbose cross-validation analysis” function in the Snipper software. From the above population genetic analysis results, it can be seen that the HZH population had close genetic relationships with EAS populations, and the structure results indicated that similar genetic structures were found in HZH and EAS populations. Therefore, we included the 57 InDel genotyping data of 206 individuals from HZH into the EAS populations for cross-validation analysis. In the cross-validation results of the five intercontinental populations (Figure 5A), individuals from EAS were correctly predicted with a probability of 0.935 3, but incorrectly predicted as SAS with a probability of 0.045 4. Individuals from the AFR were correctly predicted with a probability of 0.984 9, whereas incorrectly predicted as the AMR and EUR with probabilities of 0.007 6 and 0.007 6, respectively. The probability that an individual was correctly predicted from the AMRs was 0.665 7, while the probabilities of individuals incorrectly predicted as EAS, AFR, EUR, and SAS were 0.002 9, 0.011 5, 0.233 4, and 0.086 5, respectively. The probabilities that individuals from EUR and SAS were correctly predicted were 0.878 7 and 0.820 0, respectively. After removing the populations from SAS and AMR that showed low prediction accuracies, the prediction accuracies of the remaining three continents were significantly improved. The probabilities predicted correctly for individuals from EAS, AFR, and EUR were 0.999 1, 0.987 9, and 1, respectively (Figure 5B). The probability that individuals from EAS were incorrectly predicted to be EUR was 0.000 9.

**Figure 6:**
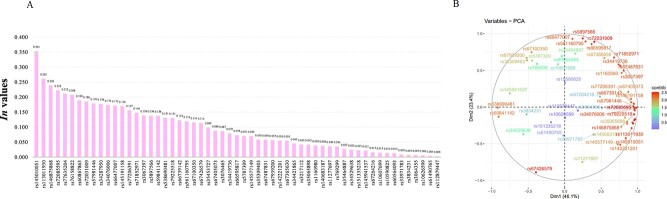
(A) *In* values of 57 InDel loci in Hezhou Han population. (B) Cos2 analyses of 57 InDel loci in Hezhou Han population.

### Informativeness values and Cos2 analyses

The population-specific divergence (PSD) values between the HZH and five intercontinental populations were performed by Snipper online software, and the calculation of the *In* value in each locus was to multiply the PSD value of the locus by 0.693 [38] (Figure 6A). The *In* value in one locus (rs145010051) was >0.3, and there were 24 loci with *In* values >0.1 and six loci <0.01. The cumulative *In* value of the 57 InDel loci was 5.523 141. Cos2 analysis was performed by *R* software based on allelic frequencies of 57 InDel loci in 31 populations, and the results were presented in Figure 6B. Due to many of loci, some of the loci near the edge of the circle did not display the names of these loci. However, it could still be seen that the loci with *In* values >0.1 were located on the periphery of the Cos2 circle, and the loci with *In* values <0.01 were around the centre of the circle [39]. These allelic frequencies of 24 loci, whose *In* values were >0.1, varied greatly in five intercontinental populations, revealing that these loci were effective in distinguishing different worldwide populations.

Based on the results of *In* values and Cos2 analyses, these 24 InDel loci with ancestral information were selected for PCA analysis. At the five intercontinental levels, individuals from HZH population scattered among populations of EAS. The AFR, EAS, and other three continents can be distinguished at the PC1 level (Supplementary Figure S5A). After removing the populations of AMR and SAS, the three intercontinental populations (AFR, EAS, and EUR) were separated from each other, and individuals from the HZH population were scattered among the EAS populations (Supplementary Figure S5B). Based on the PCA results, the genotyping data of HZH individuals were included in the EAS populations, and the genotyping data of all individuals were imported into Sinpper software for ancestry information prediction. Among the five continents, EAS, AFR, and EUR populations had forecast accuracies of 0.944 2, 0.981 8, and 0.823 1, respectively, while the AMR and SAS populations had relatively low forecast accuracies of 0.596 5 and 0.709 6 in Supplementary Figure S5C. After removing the data of the AMR and SAS populations, the prediction accuracies for the remaining three continents were all >0.98, as shown in Supplementary Figure S5D.

## Discussion

The Han nationality is widely distributed in China, and understanding the genetic characteristics and genetic structure of the Han nationality from different geographical regions is of great significance for studying the genetic backgrounds of Chinese ethnic groups. In this study, the genetic polymorphisms of 57 InDel loci in 206 HZH individuals were investigated by capillary electrophoresis, and a variety of analytical methods were used to explore the genetic relationships between the HZH and 30 reference populations. The allelic frequency distributions of the 57 InDel loci in the HZH population were relatively uniform and similar to the allelic frequency distributions in various EAS populations. All 57 InDel loci were in HWE, which indicated that the samples selected for this study were representative and could represent the entire HZH population. There were no pairwise loci which did not conform to the law of linkage equilibrium, and the multiplication principle could be used to calculate the cumulative forensic parameter values. The rs72085595 locus was relatively low forensic efficacy, while the rs5787309 locus was higher forensic efficacy. The CMP and CPE values in the HZH population were 5.5945 × 10^−24^ and 0.999 980 295, respectively, indicating that these 57 InDel loci could be used as a useful tool in individual identification and paternity testing for the HZH population. When compared with nine populations from EAS, 57 InDel loci showed large differences in forensic-related parameter values in SP and YNM populations, and the power of cumulative probability values was relatively low, but these loci can still be used as an auxiliary tool for 10 EAS populations.

Genetic distance is a method commonly used in population genetics research to evaluate the genetic relationships between multiple populations. It is a measure of genetic differences between populations or small subgroups within a population. The genetic distance between these two populations is small, demonstrating that they have a close genetic relationship or may have a common ancestor [40]. In this study, *F*_ST_ and *D*_A_ values were used to explore the genetic relationships among the HZH population and 30 reference populations around the world. The results revealed that the HZH population had the closest genetic relationships with the EAS populations and the farthest genetic relationships with the AFR populations. Among the nine reference populations from EAS, the HZH population was the closest genetic relationship with SP*.* The genetic relationships among the HZH population and the populations from EAS could also be found in the N-J tree and the circular phylogenetic tree. In addition, the MXL and PEL from AMR may be mixed populations [41, 42], so they were far away from the Native Americans. The PCA analyses at the individual and population levels highlighted the individual clustering patterns and the population genetic relationships, respectively [43]. The results demonstrated that the populations or individuals clustered according to geographic origins, and the HZH population clustered with the EAS populations. The genetic compositions of individuals within a population and the degrees of gene exchange between populations were intuitively observed in STRUCTURE analyses [44]. Regardless of the *K* values, the ancestral compositions of individuals from the HZH population were similar to those of the EAS populations, but when the *K* was 4, the SP population showed its unique ancestral composition, which was inconsistent with the EAS populations [31].

Cross-validation analyses of five intercontinental populations signified that the individuals from EAS and AFR both had prediction accuracies above 0.9; both individuals from EUR and SAS were accurately predicted to be more than 0.8, while the individuals from the AMR population had prediction accuracies below 0.7. After removing the genotyping data of SAS and AMR, individuals from these three continents (EAS, EUR, and AFR) were correctly predicted with probabilities >0.95. The original intention of this 60 InDel multiplex system was to carry out individual identifications in EAS populations, which may lead to poor ancestry inference performance of this system and could not accurately distinguish differentindividuals from five intercontinental populations. However, this system had a certain discriminative power for identifying intercontinental origins of the individuals from three intercontinental populations, and it also indicated that there were InDel loci in this system which could be explored for ancestry inference efficiency.

The comparison of allele frequencies among the 31 populations from five continents displayed that there were large differences in allelic frequencies of some loci among the EAS, EUR, and AFR populations, so it was speculated that these loci may be used as ancestral information inference loci. The *In* value is a parameter commonly used in population genetics to evaluate whether genetic markers have ancestral inference power. It can be used to assess the degrees of genetic differentiations among populations, and genetic markers exhibiting higher *In* values are helpful in distinguishing this population from other populations [38]. Therefore, the Snipper software was used to calculate the *In* value of each locus between the HZH population and five intercontinental populations. The 24 InDel loci with *In* values >0.1 had large allele frequency differences between EAS and the other four intercontinental populations. In addition, we also performed Cos2 analysis using *R* software based on the frequencies of the insertion alleles in 31 populations. In the Cos2 plot, the loci closer to the circle have greater allelic frequency differences among different populations. The 24 loci with *In* values >0.1 were located near the circle. In order to verify the ancestry inference power of these 24 loci, PCA analyses of 31 populations were performed. The results demonstrated that individuals from the HZH population were scattered among the populations from EAS, and these 24 loci were able to distinguish different individuals from EAS, AFR, and EUR populations. In the cross-validation analyses of five continents, the prediction accuracies for SAS and AMR populations were <0.8, the prediction accuracies of the remaining three continents were >0.8. In addition, in the cross-validation analyses of the three continents, the prediction accuracies of EAS, AFR, and EUR were all >0.98. It could be concluded that these 24 InDel loci could effectively infer the origins of EAS, AFR, and EUR populations, and they had high ancestry inference abilities. In addition to being used for individual identification research, the system could also use the 24 InDel loci to assist in the inference of the intercontinental origins of criminal suspects. However, the accurate inferences of individual origins from two intercontinental populations (AMR and SAS) cannot be achieved, so it is still necessary to explore more ancestral inference loci in future research to achieve the high effect of accurate differentiations among different populations from five continents or even different groups within the same continent. In addition, in order to ensure compatibility with the reagent system, these 24 InDel loci can be considered as candidate loci in future ancestry inference research.

## Conclusion

In this study, the genotyping data of 57 autosomal InDels and two Y-InDel in the HZH population were successfully obtained, and the allelic frequencies and forensic-related parameters of each locus were calculated according to the relevant formula. The CPM and CPE values indicated that the AGCU InDel 60 panel can be used as a powerful tool for individual identification and paternity testing in the HZH population. Then, the genetic relationships between the HZH population and 30 worldwide populations were explored using multiple analytical methods. The HZH population had closer genetic relationships with EAS populations and further genetic relationships with the AFR populations. The results of two prediction methods manifested that genotyping data of the 57 InDel loci could more accurately predict whether unknown individuals originated from EAS, EUR, and AFR populations. In addition, 24 out of the 57 InDel loci with ancestral inference potential can also achieve the purpose of distinguishing individual origins from EAS, EUR, and AFR.

## Supplementary Material

Supplementary_material_owae021

Supplementary_figure_legends_owae021
