# Supplementary figures and images for: Comprehensive elucidation on the genetic profile of the Hezhou Han population via an efficient InDel panel
Source: Forensic Sci Res. 2024 Apr 9;10(1):owae021. doi: 10.1093/fsr/owae021 (PMC11850652; doi:10.1093/fsr/owae021)

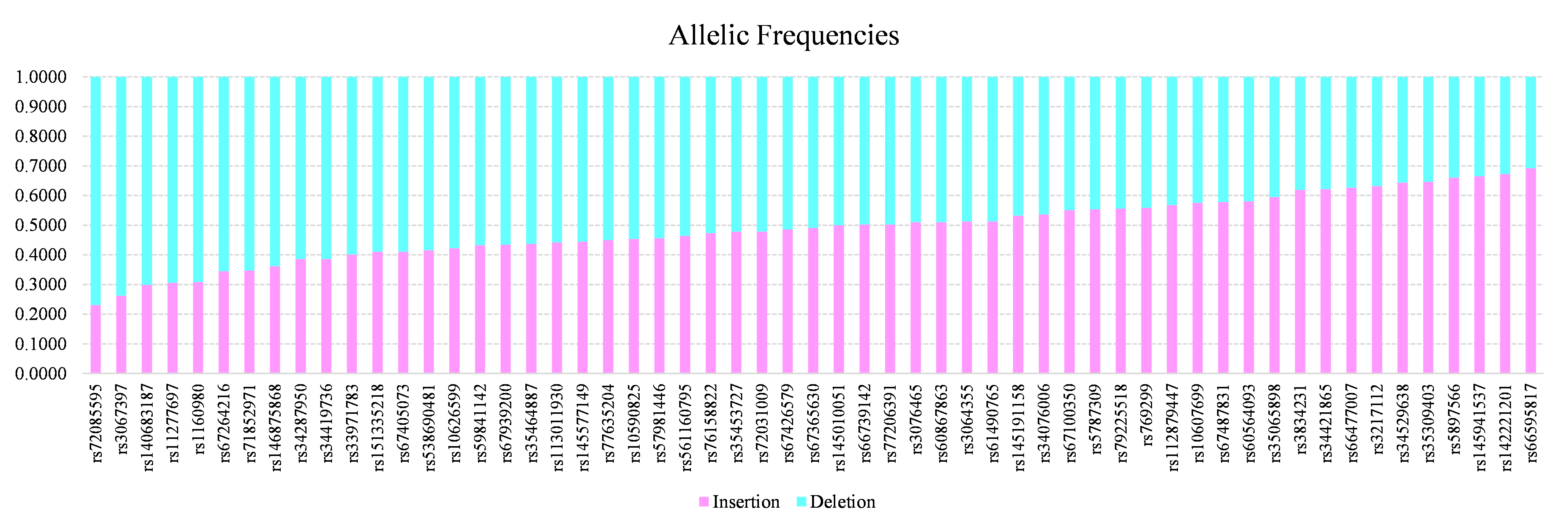

Supplement: Supplementary_material_owae021 [file supplementary_material_owae021.zip › Supplementary Figure 1 .tif]

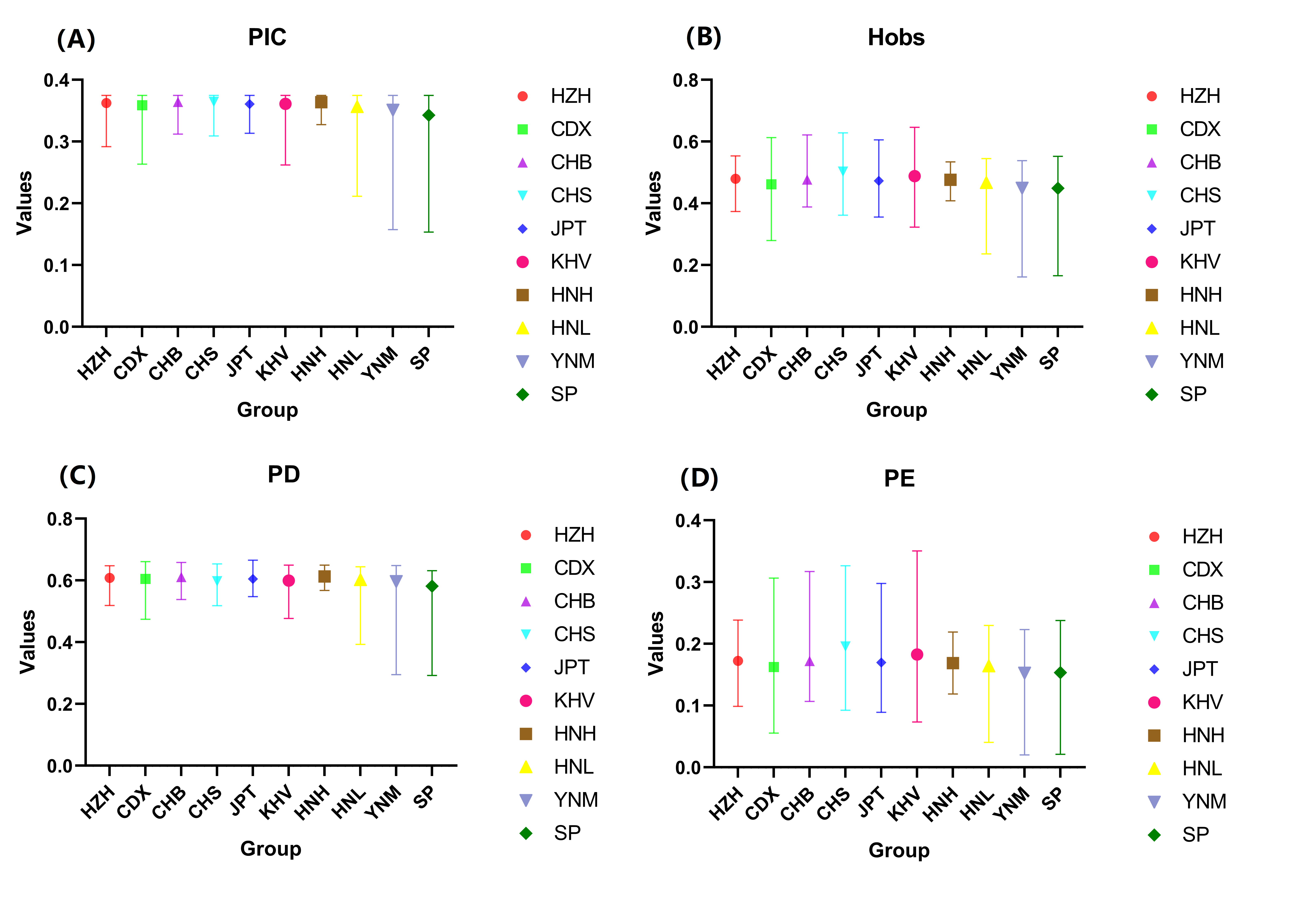

Supplement: Supplementary_material_owae021 [file supplementary_material_owae021.zip › Supplementary Figure 2.tif]

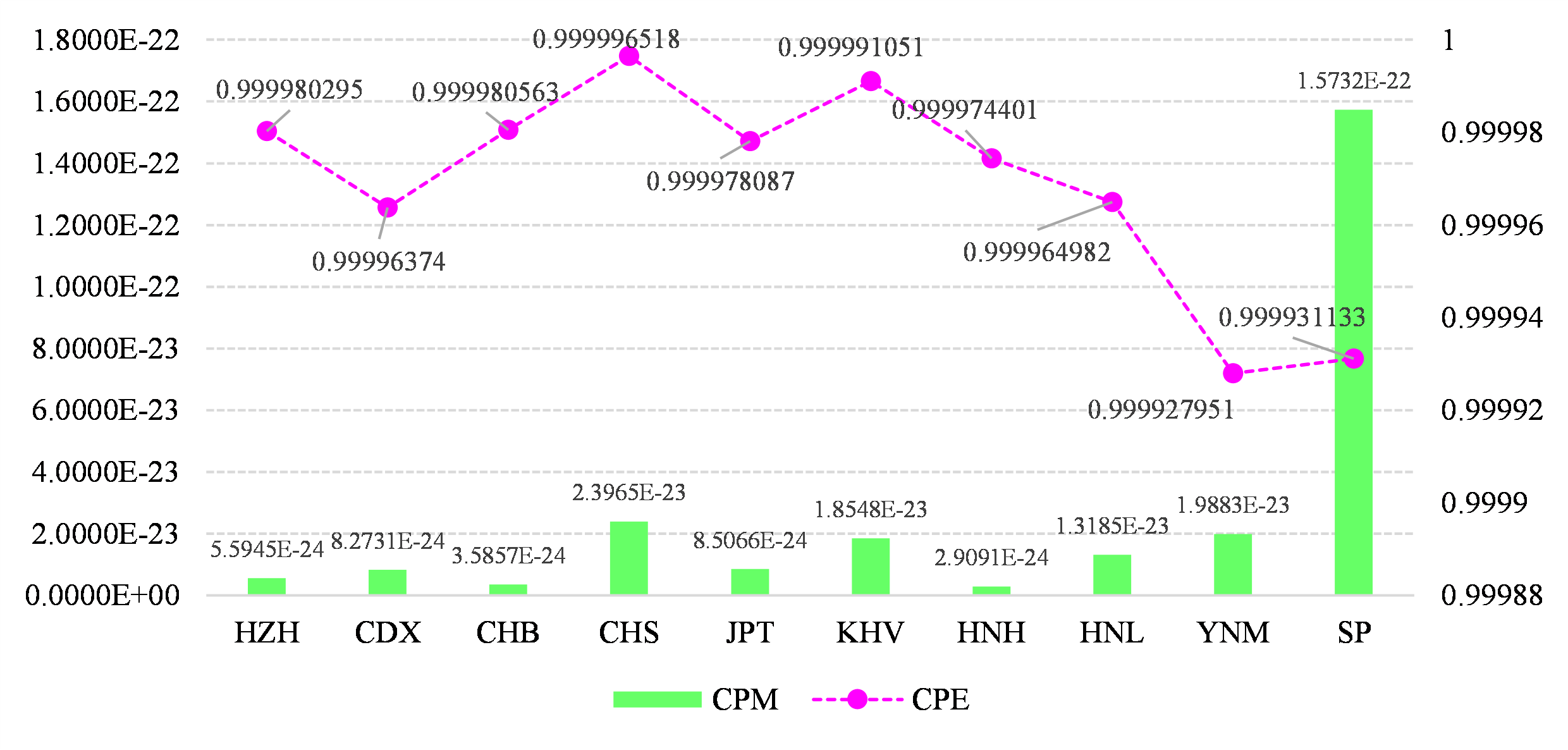

Supplement: Supplementary_material_owae021 [file supplementary_material_owae021.zip › Supplementary Figure 3.tif]

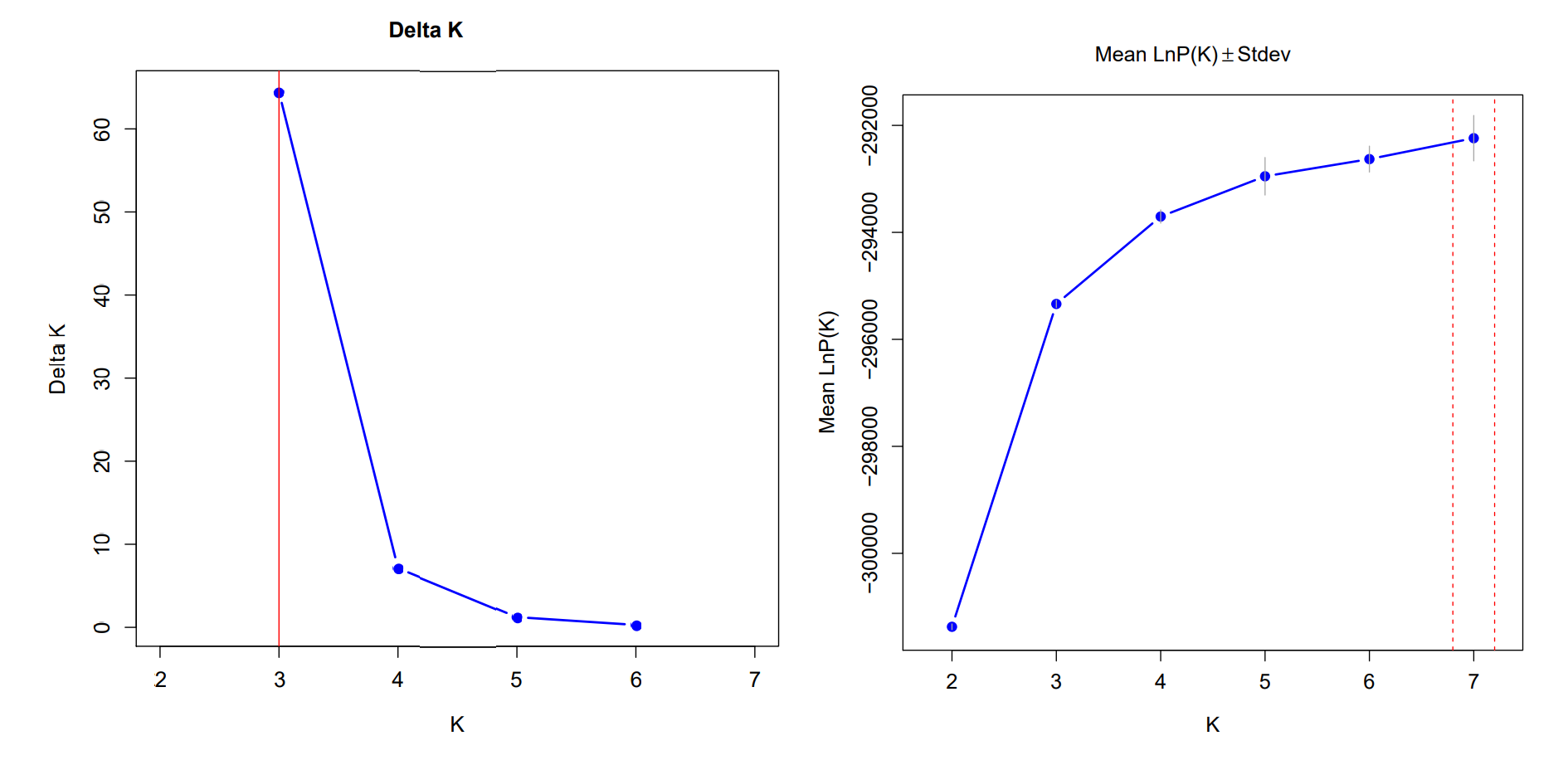

Supplement: Supplementary_material_owae021 [file supplementary_material_owae021.zip › Supplementary Figure 4.tif]

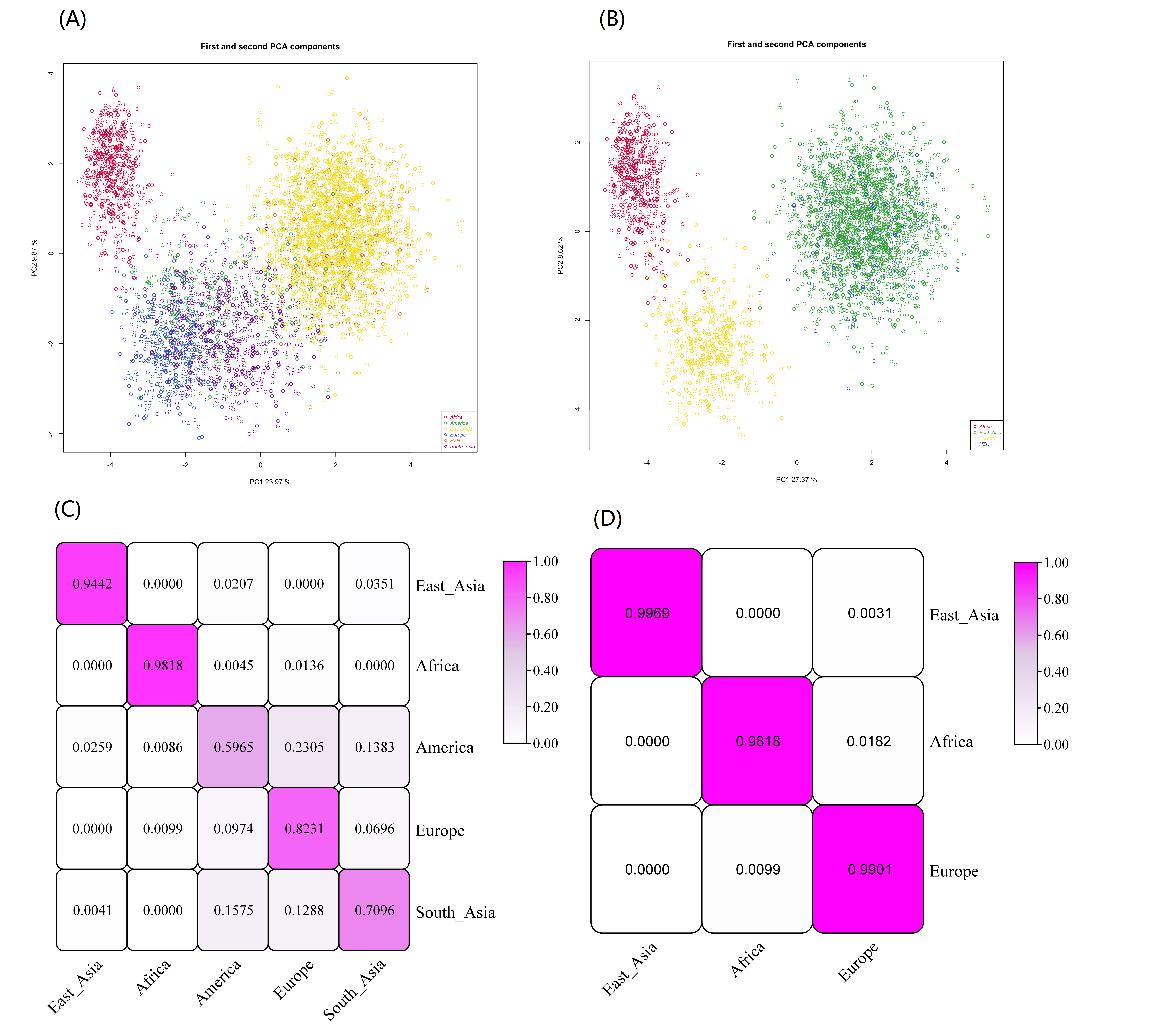

Supplement: Supplementary_material_owae021 [file supplementary_material_owae021.zip › Supplementary Figure 5.tif]
