# Supplementary material for: Comprehensive elucidation on the genetic profile of the Hezhou Han population via an efficient InDel panel
Source: Forensic Sci Res. 2024 Apr 9;10(1):owae021. doi: 10.1093/fsr/owae021 (PMC11850652; doi:10.1093/fsr/owae021)
Supplement: Supplementary_figure_legends_owae021 [file supplementary_figure_legends_owae021.docx]

**Supplementary figure legends**

**Supplementary figure 1** Allelic frequencies of 57 InDel loci in Hezhou Han population.

**Supplementary figure 2** Comparisons of four forensic parameter values for ten populations. (A) Polymorphism information content (PIC); (B) Observed heterozygosity (Hobs); (C) Power of discrimination (PD); (D) Exclusion probability (PE).

**Supplementary figure 3** Cumulative match probability (CPM) and combined exclusion probability (CPE) values for 57 InDel loci in ten populations from East Asia.

**Supplementary figure 4** Determining the line chart with the optimal K of three. (A) Delta K. (B)Mean LnP(K) ± Stdev.

**Supplementary figure 5** PCA analyses and cross-validation analyses of ancestral origin inference. (A) PCA analyses among Hezhou Han population and five intercontinental populations. (B) PCA analyses among Hezhou Han population and three intercontinental populations. (C) Cross-validation analyses of five intercontinental populations. (D) Cross-validation analyses of three intercontinental populations.
